# Evaluation of flagellum-related proteins FliD and FspA as subunit vaccines against *Campylobacter jejuni* colonisation in chickens

**DOI:** 10.1016/j.vaccine.2016.02.052

**Published:** 2016-04-04

**Authors:** C. Chintoan-Uta, R.L. Cassady-Cain, M.P. Stevens

**Affiliations:** The Roslin Institute and The Royal (Dick) School of Veterinary Studies, University of Edinburgh, Easter Bush, Midlothian EH25 9RG, UK

**Keywords:** *Campylobacter jejuni*, Chickens, Subunit, Vaccine, Flagellar cap protein, Flagellum secreted protein

## Abstract

*Campylobacter* is the leading cause of food-borne diarrhoea in humans in the developed world and consumption of contaminated poultry meat is the main source of infection. Vaccination of broilers could reduce carcass contamination and zoonotic infections. Towards this aim, we evaluated recombinant anti-*Campylobacter* subunit vaccines based on the flagellum-capping protein FliD and the flagellum-secreted protein FspA as they are immunogenic in chickens and the flagellum is vital for colonisation. In three studies, a recombinant FliD vaccine induced a transient but reproducible and statistically significant decrease of c. 2 log_10_ CFU/g in caecal colonisation levels at 49 days post-primary vaccination on the day of hatch. Levels of serum IgY specific to FliD positively correlated with caecal bacterial counts in individual birds, indicating that such antibodies may not play a role in protection. The data add to the limited repertoire of candidate antigens for the control of a key foodborne zoonosis.

## 1 Introduction

*Campylobacter* is the leading cause of food-borne diarrhoeal disease in the developed world and the European Food Standards Agency has predicted that nine million cases of human campylobacteriosis occur every year across EU27 countries, resulting in 0.35 million disability-adjusted life years per annum and a cost of €2.4 billion [1]. Source attribution studies show that up to 80% of human cases may be linked to consumption or handling of contaminated poultry meat [1]. A reduction in carcass contamination of 2 log_10_ CFU/g of caecal contents has been predicted to result in a decrease of up to 30-fold in cases of human infection due to poultry [2], however few effective treatments or vaccines have been described. Given that the latest UK-wide surveys reported contamination of 73% of raw chicken on sale during the 2014–2015 year [3] and an estimated 685,000 human infections in 2013 [4], there is a compelling case for vaccination of poultry at the farm level.

Previous vaccination studies have evaluated flagellin subunits as the flagellum is vital for colonisation of the avian intestines [5], [6], [7]. The flagellin-based vaccines showed variable levels of protection and failed to induce heterologous protection, possibly due to glycosylation of flagella which can vary with the strain [8], [9] and phase variation [10]. In order to address these limitations we tested two other flagellum-related proteins as recombinant purified subunit vaccines: FliD, the flagellar cap protein [11], a 70 kD protein with a minimum protein sequence conservation of 91% identity across the *Campylobacter jejuni* entries available in National Centre for Biotechnology Information's database and FspA, a flagellum secreted protein [12] with a molecular weight of 16.5 kD and a minimum protein conservation of 97%. FliD is known to be immunogenic following natural infection of *Campylobacter* in broilers [13] and is one of the proteins recognised by maternal antibodies transferred in the egg [14]. Maternal antibodies are thought to delay colonisation of chicks by *Campylobacter* in the first few weeks of life [15]. FspA has been demonstrated to be protective against clinical signs and colonisation in a mouse vaccination and challenge model of *C. jejuni* infection [16].

This report describes the development and testing of FliD and FspA subunit vaccines and demonstrates that FliD, but not FspA, induces transient but statistically significant and reproducible protection in vaccinated chickens. This study adds a novel antigen to the limited repertoire of protective candidates for control of *Campylobacter* in the avian reservoir.

## 2 Methods

Bacterial culture was carried out as described previously [18]. Strains used are detailed in Table 1. Expression vectors for production of *C. jejuni* strain M1 CjaA, FliD and FspA as C-terminal fusions to glutathione-S-transferase (GST) were prepared by ligation of restriction endonuclease-digested PCR amplicons with plasmid pGEX-4T1 essentially as we described [19]. Plasmid constructs and the primers and enzymes used for their production are given in Table 1. Constructs were verified by dideoxy chain-termination sequencing (Source Bioscience, UK) and transformed in *Escherichia coli* Rosetta (Novagen, UK) for expression of the recombinant proteins. Strains were grown at either 28 °C for expression from pGEX-4T1-*cjaA* and pGEX-4T1-*fliD* or 37 °C for expression from pGEX-4T1 and pGEX-4T1-*fspA*. Cultures were induced with either 0.1 mM isopropyl β-d-1-thiogalactopyranoside (IPTG; pGEX-4T1-*cjaA* and pGEX-4T1-*fliD*) or 1 mM IPTG (pGEX-4T1 and pGEX-4T1-*fspA*). Purification was undertaken as described [18] in batch format using glutathione sepharose beads (GE Lifesciences, UK) following manufacturer's protocol with elution in 40 mM glutathione.

Protein preparations were validated by Western blotting using a rabbit anti-GST antibody (Santa Cruz Biotechnology, USA) at 1:10,000 dilution and bound antibody was detected with an HRP-conjugated anti-rabbit IgG (Sigma–Aldrich, UK) at 1:10,000 dilution.

Once validated, the purified *Campylobacter* antigens were tested for their protective efficacy in chickens. All procedures were conducted under Home Office licence 60/4420, according to the requirements of the Animal (Scientific Procedures) Act 1986, and with the approval of the local ethical review committee. The line of birds, adjuvant and the schedule of vaccination and challenge were as described [18] to permit comparison with earlier work. A total of 240 White Leghorn chickens were used and obtained on the day of hatch from a Home Office licensed breeding establishment. Briefly, three separate studies were conducted, each with the same design, including vaccination with GST or GST-CjaA as negative and positive controls, respectively. Birds were given the primary vaccination on the day of hatch, a booster 14 days later and challenged with 10^7^ CFU of *C. jejuni* M1 at 28 days post-hatch (dph). For each vaccination, birds received 4.3 × 10^−10^ moles of recombinant protein for parity with previous studies [18], [19]. The antigens were mixed 1:1 with TiterMax Gold adjuvant (Sigma Aldrich, UK) and delivered in two subcutaneous injections of 50 μl each on the thorax using a 21 g 1″ needle. *Campylobacter* enumeration was performed *post-mortem* at weekly intervals following challenge and samples of blood collected for measurement of humoral responses.

At the end of each vaccination trial, enzyme-linked immunosorbent assays (ELISAs) were carried out to measure antigen-specific serum IgY against FliD, FspA and CjaA, as described [18]. Coating conditions were optimised using chequerboard analyses for each antigen. Coating conditions were as follows: 0.5 μg/ml GST-CjaA, 1 μg/ml GST-FliD and 1 μg/ml GST-FspA. Serum was diluted 1:500 in all ELISAs.

Statistical analyses were performed using Minitab 17 (Minitab, UK). A general linear model (GLM) and *post hoc* Dunnet's tests were used to test for differences in caecal colonisation. A two-sided *t*-test was used to assess changes in serum IgY compared to unvaccinated controls only at each time-point. *P* values of ≤0.05 were considered significant.

## 3 Results

**Fig. 1:**
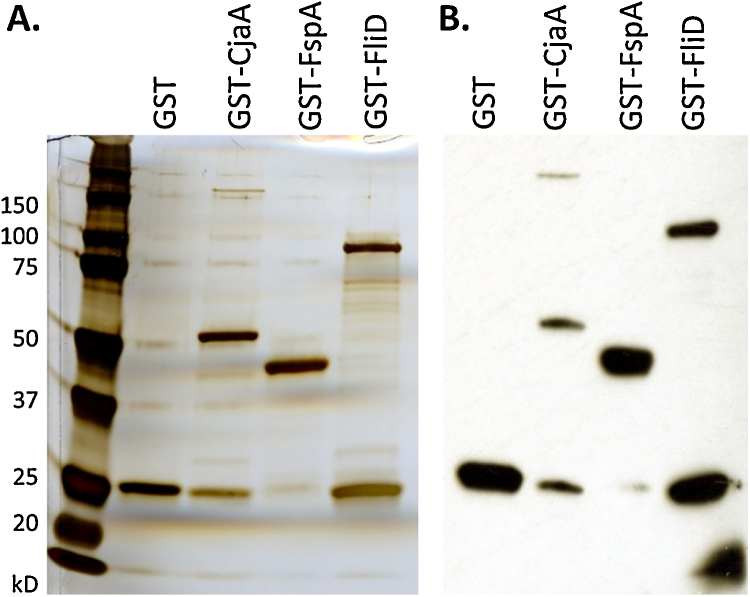
GST-tagged recombinant *Campylobacter* antigens used in vaccination experiments in chickens. (A) Silver staining of typical preparations of GST-tagged *Campylobacter* antigens used in vaccination experiments in chickens. (B) Purity of the protein preparations shown in panel A assessed *via* a Western blot with an anti-GST antibody.

Typical preparations of antigens used for vaccination are shown in Fig. 1A. Separate preparations of GST-FliD and GST-CjaA were used for each vaccination trial and the same preparation of GST-FspA and GST were used for all trials. In Western blots, the GST and GST-FspA preparations gave a single species, whereas the GST-CjaA and GST-FliD preparations contained a truncated variant corresponding to the size of GST as well as the dominant fusion protein (Fig. 1B), as observed previously [18]. This may have inadvertently resulted in administration of a lower molar quantity of the FliD and CjaA antigens relative to other fusion proteins.

**Fig. 2:**
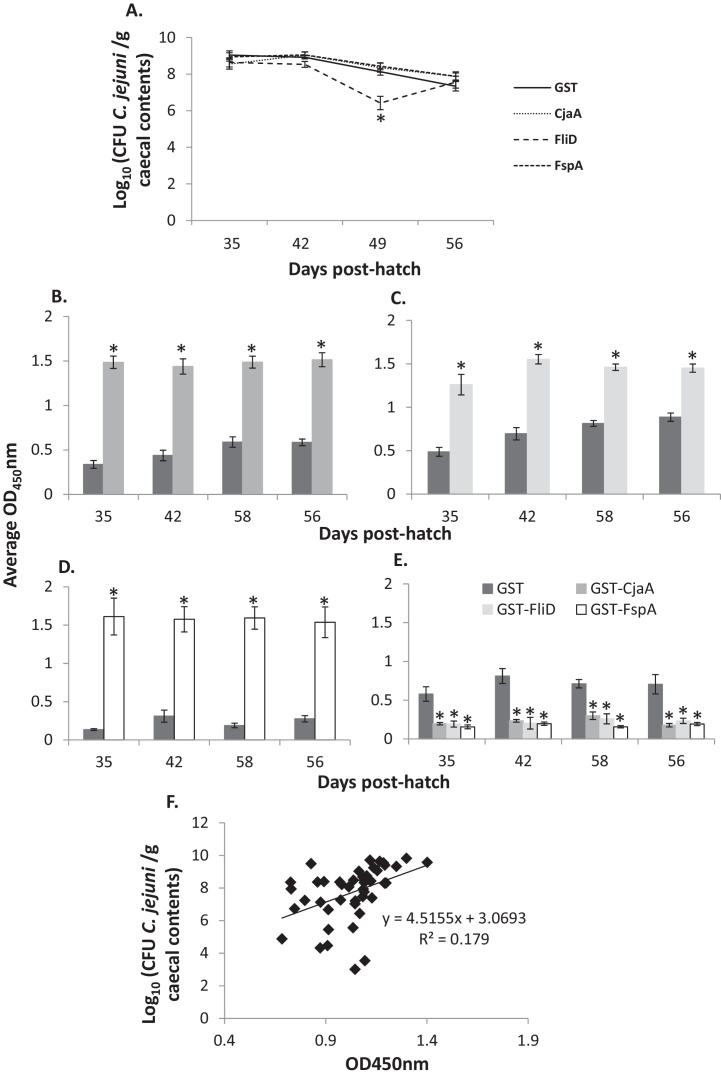
*Campylobacter* caecal colonisation levels and the induction of antigen-specific serum IgY following vaccination of chickens with recombinant GST-tagged *Campylobacter* antigens. (A) Levels of *C. jejuni* M1 colonisation in the caeca of vaccinated chickens, sampled at weekly intervals at *post-mortem* examination. The lines show an inferred course of infection and not actual kinetics. Between 6 and 20 birds were sampled per week per treatment group. Data for FspA derive from two independent replicates whereas the other groups were tested in three independent trials. (B–D) Reactivity of sera collected from GST and GST-CjaA, GST-FspA and GST-FliD vaccinated birds to the respective GST-tagged *Campylobacter* antigens expressed as average OD_450nm_. (E) Reactivity of sera collected from each group of vaccinated birds to GST only expressed as average OD_450nm_. For panels B–E error bars represent the standard error of the mean (SEM) and asterisks denote statistical significance at *P* < 0.05. (F) Linear regression of caecal *Campylobacter* counts on OD_450nm_ readings obtained in ELISAs measuring reactivity against GST-FliD in individual birds in the GST-FliD vaccinated group.

Following vaccination and homologous challenge with *C. jejuni* in chickens, differences in caecal colonisation were analysed with a second order hierarchical GLM taking into account interactions between treatment group and time of sampling (*R*^2^ = 0.41). When compared to GST-vaccinated controls, averaged across two biological replicates, GST-FspA failed to induce statistically significant reductions at any of the time intervals sampled (Fig. 2A). In contrast, in chickens vaccinated with GST-FliD a significantly different course of caecal *Campylobacter* colonisation was observed across three independent studies, compared to both the GST (*P* < 0.001) and GST-CjaA (*P* < 0.001) vaccinated groups (Fig. 2A). *Post hoc* Dunnet's tests indicated that this was due to a significant and reproducible reduction present only at 49 days post-hatch (*P* < 0.001 when compared to both GST and GST-CjaA). A reduction in caecal colonisation was absent one week later but the timing and magnitude of the trend were consistently observed across each of three vaccination trials. As previously observed, GST-CjaA proved not to be protective [18], however, this is in contrast to our previous studies using histidine-tagged CjaA in a different chicken line [19].

ELISA measurements of serum antigen-specific IgY in vaccinated birds were used to assess immunogenicity of the vaccines and whether antibody levels correlated with *Campylobacter* colonisation. A significantly higher level of antigen-specific serum IgY was detected in all vaccinated groups compared to the group vaccinated with GST only at all time intervals sampled (Fig. 2B–E), indicating successful delivery and priming. However, in the GST-FliD vaccinated group, the kinetics of the serum IgY responses induced by vaccination did not correspond to the time when reductions in caecal *Campylobacter* numbers were observed. Furthermore, a positive correlation was observed between the magnitude of the antibody response and caecal *Campylobacter* counts in individual birds in the GST-FliD vaccinated group (*R*^2^ = 0.18; *P* = 0.004; Fig. 2F). This suggests that anti-FliD antibodies are unlikely to be associated with the protection observed. Furthermore these antibodies failed to agglutinate *C. jejuni in vitro* or prevent bacterial motility in a soft agar diffusion test (data not shown).

## 4 Discussion

If widely applied, a vaccine that prevents or reduces *Campylobacter* colonisation of poultry could control human infection at its primary source. To circumvent the limitations of flagellin-based vaccines mentioned above, we tested the efficacy of purified recombinant GST-FliD and GST-FspA, two flagellum-related proteins, in reducing caecal *Campylobacter* colonisation. In contrast to studies in mice using a 6xHis-tagged recombinant vaccine [16], no protection was observed using GST-FspA in chickens. This could be due to differences in the affinity tag used for purification, dose of the vaccine or adjuvant used in this study or due to differences in the role of FspA in *Campylobacter* infection in chickens and mice. Further work would be needed to determine the basis of this discrepancy.

Even though the FliD-based vaccine produced a c. 2 log_10_ CFU/g reduction predicted by modelling to have an impact on human infections [2], the transient nature of protection, high cost of production and need for repeated manual doses precludes commercial usefulness. Protection may differ if evaluated in a low dose challenge model involving contaminated litter or seeder birds. The short-lived protection may be due to immune evasion by *C. jejuni* involving altered expression of FliD or the flagellum. It is known that both expression [20] and glycosylation of flagella is phase variable [10], however loss of motility may be expected to be attenuating in the chicken unless other adaptations occur. Diminished protection could also be due to waning of the protective immune response(s), and further work is needed to define the nature of cell-mediated responses to vaccination and their association with protection. The lack of protection conferred by vaccination with GST-CjaA was recently reported [18] and may reflect use of a different fusion protein, molar dose, adjuvant and bird line in the current study compared to previous work [19]. The GST affinity tag was chosen as it has been claimed to produce higher yield and purity proteins compared to the 6xHis tag and previous studies demonstrated protection against *Campylobacter* using other GST-tagged proteins [18]. However, its use may have resulted in conformational changes in the case of CjaA which may account for its lack of protection relative to the His-tagged protein used elsewhere [19]. Despite the difference in protection observed with FliD- and FspA-based vaccines, both antigens induced significant increases in antigen-specific serum IgY levels compared to GST-only vaccinated control birds. This discrepancy and the positive correlation observed between levels of anti-GST-FliD serum IgY and caecal *Campylobacter* count in individual birds suggests that antibody may not play a central role in protection, accepting that only IgY was measured here and studies with Ig knockout birds are required to formally exclude a role for antibody. Findings for the FliD-based vaccine are consistent with our recent observations using a SodB-based subunit vaccine which was protective despite an apparent lack of surface expression of SodB in *C. jejuni* [18]. Nevertheless our study adds FliD to the limited repertoire of protective antigens described to date, accepting that efficacy is modest, transient and unlikely to be suitable for commercial vaccines without significant further refinement.

## Figures and Tables

**Table 1:** Bacterial strains, plasmids and primers used in this study.

Strain or plasmid	Description	Source or reference	Use	Primers used for construction^a^
*E. coli* XL1 Blue	F^−^ (f80d*lac*ZDM15) D(*lac*ZYA-*argF*)*U169 recAl endAl* *hsdRl7*(rk − mk + ) *supE44 l*^*−*^*thi-l gyrA relA*	Invitrogen, UK	Production and propagation of plasmid constructs.	N/A
*E. coli* Rosetta pLysE BL21 (DE3)	F^−^*ompT hsdSB(r*_*B*_^*−*^*, m*_*B*_^*−*^*) dcm galλ (DE3)*	Novagen, UK	Expression of GST-tagged proteins	N/A
*C. jejuni* M1	Wild-type human isolate	[17]	Source of gDNA for cloning of antigens and model strain for challenge of chickens	N/A
pGEX-4T1	Vector for expression of recombinant proteins fused to the C terminus of GST, under a *lac* promoter	GE Lifesciences, UK	Expression of GST-tagged *Campylobacter* antigens and of GST alone.	N/A
pGEX-4T1-*cjaA*	*C. jejuni* M1 *cjaA* fused 3′ to GST in pGEX-4T1	This study	Expression of GST-tagged CjaA	Fwd: 5′CGCGCGGGATCCATGAAAAAAATACTTCTAAG3'′ Rev: 5′GCGCGCGGCCGCTTAAATTTTTCCACCTTCAA3′
pGEX-4T1-*fliD*	*C. jejuni* M1 *fliD* fused 3′ to GST in pGEX-4T1	This study	Expression of GST-tagged FliD	Fwd: 5′CGCGCGGGATCCATGGCATTTGGTAGTCTATC3′ Rev: 5′GCGCGCGGCCGCTTAATTATTAGAATTGTTTG3′
pGEX-4T1-*fspA*	*C. jejuni* M1 *fspA* fused 3′ to GST in pGEX-4T1	This study	Expression of GST-tagged FspA	Fwd: 5′CGCGCGGGATCCATGCAAATTAACAATTCCTT3′ Rev: 5′CGCGCGCGGCCGCTCAAGCTTGTTGGCTTTGGA3′

a Underlined sequences represent sequences recognised by the *Bam*HI and *Not*I restriction enzymes used for cloning.
